# Extracellular vesicle analysis of plasma allows differential diagnosis of atypical pancreatic serous cystadenoma

**DOI:** 10.1038/s41598-023-37966-5

**Published:** 2023-07-06

**Authors:** Katherine S. Yang, Aileen O’Shea, Piotr Zelga, Andrew S. Liss, Carlos Fernandez Del Castillo, Ralph Weissleder

**Affiliations:** 1 Center for Systems Biology, Massachusetts General Hospital, 185 Cambridge St, CPZN 5206, Boston, MA 02114 USA; 2 Department of Radiology, Massachusetts General Hospital, 32 Fruit St, Boston, MA 02114 USA; 3 Department of Surgery, Massachusetts General Hospital, 32 Fruit St, Boston, MA 02114 USA; 4 Department of Systems Biology, Harvard Medical School, 200 Longwood Ave, Boston, MA 02115 USA

**Keywords:** Diagnostic markers, Gastrointestinal diseases

## Abstract

Increased use of cross-sectional imaging has resulted in frequent detection of incidental cystic pancreatic lesions. Serous cystadenomas (SCAs) are benign cysts that do not require surgical intervention unless symptomatic. Unfortunately, up to half of SCAs do not have typical imaging findings (“atypical SCAs”), overlap with potentially malignant precursor lesions, and thus pose a diagnostic challenge. We tested whether the analysis of circulating extracellular vesicle (EV) biomarkers using a digital EV screening technology (DEST) could enhance the discrimination of cystic pancreatic lesions and avoid unnecessary surgical intervention in these atypical SCAs. Analysis of 25 different protein biomarkers in plasma EV from 68 patients identified a putative biomarker signature of Das-1, Vimentin, Chromogranin A, and CAIX with high discriminatory power (AUC of 0.99). Analysis of plasma EV for multiplexed markers may thus be helpful in clinical decision-making.

## Introduction

The expanding use of cross-sectional imaging has increased the detection of cystic pancreatic lesions^1^. Serous cystadenomas (SCAs), mucinous cystadenomas (MCNs), and intraductal papillary mucinous neoplasms (IPMNs) comprise the majority of cystic neoplasms encountered in clinical practice^2,3^. Serous cystadenomas of the pancreas (SCAs) are considered benign, are mostly asymptomatic, and have minimal risk of malignant transformation^4^. Accordingly, incidentally-discovered SCAs do not warrant surgical intervention unless there is diagnostic uncertainty^3,5^ or cysts become symptomatic.

**Figure 1:**
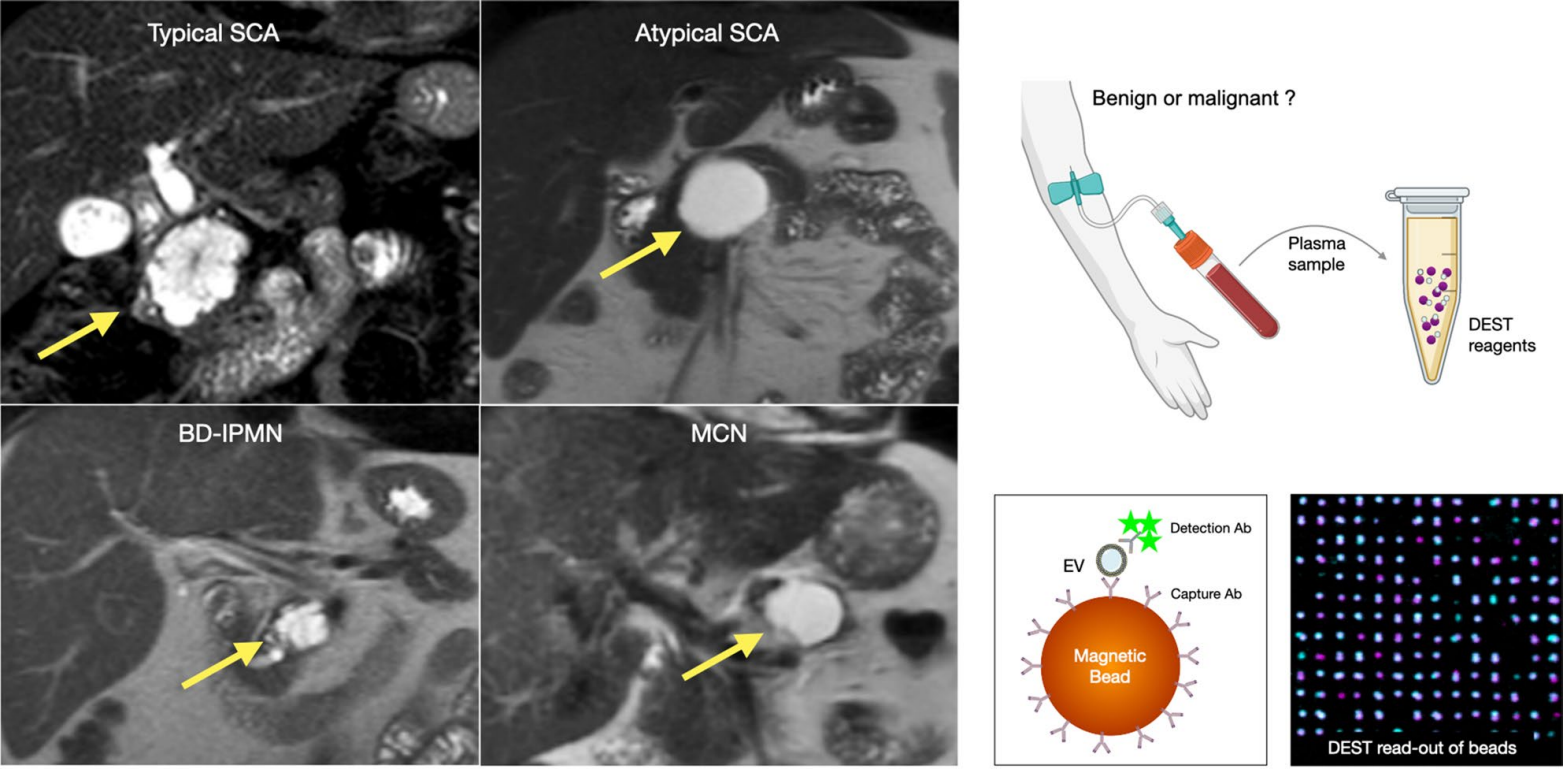
Differential diagnosis of atypical SCA. Atypical SCA can be mistaken for other pancreatic cysts (MCN, BD-IPMN), some of which have malignant potential (left panel). Plasma EV analysis using a digital EV screening technology (DEST, right) represents a new approach for the identification and management of atypical SCA. The DEST assay measures EV biomarkers using a magnetic bead amplification technique. EV containing a biomarker of interest are captured on an antibody-coated bead, thus concentrating EV magnetically. Biotinylated detection antibodies and subsequent tyramide signal amplification is used to read out positive beads by imaging (illustrative example bottom right) or flow cytometry (readout for this manuscript). An example of positive DEST beads is shown in the lower right, with single beads colored purple and positive beads from tyramide signal amplification colored cyan. Images were generated using ImageJ2, version 2.9.0/1.53t (https://imagej.net/software/fiji/).

Classical imaging appearances of macrocystic SCAs include a multilocular cystic lesion with a central scar or a microcystic lesion with well-defined borders^6^. However, these appearances are estimated to be absent in a third to a half of cases^6–8^. Atypical appearing SCAs, usually unilocular lesions, can share imaging characteristics of MCNs and IPMNs^9^ (Fig. 1). In contradistinction to SCAs, MCNs and IPMNs harbor malignant potential, representing an adenoma-carcinoma sequence^10^. While asymptomatic and incidentally detected SCAs do not warrant surgical intervention, the treatment paradigm for cystic neoplasms with malignant potential differs. Surgical intervention for these lesions carries risks. Although mortality for pancreatic resection has decreased and is now under 3% in high-volume centers^3^, the major complication rate is still high, ranging between 0.9 and 14.4%^3^.

Accurate diagnostic evaluation of atypical cystic pancreatic neoplasms remains limited to this date. A potential strategy for discriminating between different lesions is molecular profiling of blood (“liquid biopsy”). The analysis of extracellular vesicles (EV) in plasma, has gained much interest as it is believed that most malignant lesions shed vesicles at increased rates compared to non-malignant cells^11^. In contradistinction to biopsy specimens, which sample a relatively small fraction of the total tumor, EV analysis can provide an overview of lesion heterogeneity. To function as an effective adjunct diagnostic tool, assays for EV detection must possess high sensitivity early in the course of the disease at a low tumor volume^12^. For this reason, digital techniques for EV analysis have gained considerable interest. One such method, digital extracellular vesicle screening technology (DEST), uses a bead-based amplification to result in a 10,000-fold increased sensitivity compared to ELISA in an assay that can be completed within 3 h and is thus well suited for clinical testing^13^ (Fig. 1, S1). Unlike existing digital assays, such as ddPCR^14^, DEST offers several advantages for clinical applications. It is less technically demanding, faster, more cost-effective, compatible with common lab equipment, and more consistent than ddPCR, as it does not rely on droplet formation. Here, we developed a DEST method for 16 new EV protein biomarkers, bringing the total to 25 EV markers in combination with our previous study^13^ (Fig. S1). Using this validated method, we determined whether DEST analysis of blood or cyst fluid could enhance diagnostic discrimination of cystic pancreatic lesions identified by imaging and ultimately obviate the need for surgical intervention in atypical SCAs.

## Results

### DEST analysis for EV signature of cystic lesions

To explore the utility of a blood-based EV test for pancreatic lesions, we first performed a comprehensive literature search to summarize protein biomarkers previously associated with different pancreatic tumors or cystic lesions. We started with reported or otherwise cyst-related biomarkers identified by literature^15–24^. For each biomarker, we identified capture and detection antibody pairs. Antibodies were first validated on negative and positive controls from purified cell-line derived EV, cell lysates, or purified proteins. Glycogen was detected using the glycogen-binding protein STBD1^25,26^ (Fig. S2). Tested biomarkers are putatively associated with EV either based on our studies with cell line-derived EV or previous studies reported in the literature. Of these, 16 EV biomarkers that are putatively associated with SCA either as positive or negative biomarkers passed the quality control steps and were used: VEGFA, GLUT1, HIF1α, MUC6, p53, pan-Cytokeratin, VEGF/PIGF, Vimentin, Inhibin, Chromogranin A, VEGFC, Carbonic Anhydrase IX, VHL, Calponin 1, Glycogen, and Cytokeratin 18.

We initially tested EV from a broad spectrum of patients with “cystic lesions of the pancreas” as the unifying theme because that is how patients present clinically. To represent the most common clinical spectrum, we included SCA (15), low-grade (benign) and high-grade (pre-malignant) IPMN (23), HC (8), MCNs (4), other cysts (8), and cystic pancreatic adenocarcinoma (10) (Fig. S3). Subsequently, a subset analysis was done on the 38 patients with (a)typical SCA and/or IPMN. All samples were examined for the presence of 16 putative SCA biomarkers. All patients with focal pancreatic lesions underwent surgery enabling accurate diagnosis of lesions. Visual inspection of the raw data showed no single EV biomarker consistently positive for SCAs (Fig. S4).

### EV analysis allows the detection of invasive cancers in cystic lesions

**Figure 2:**
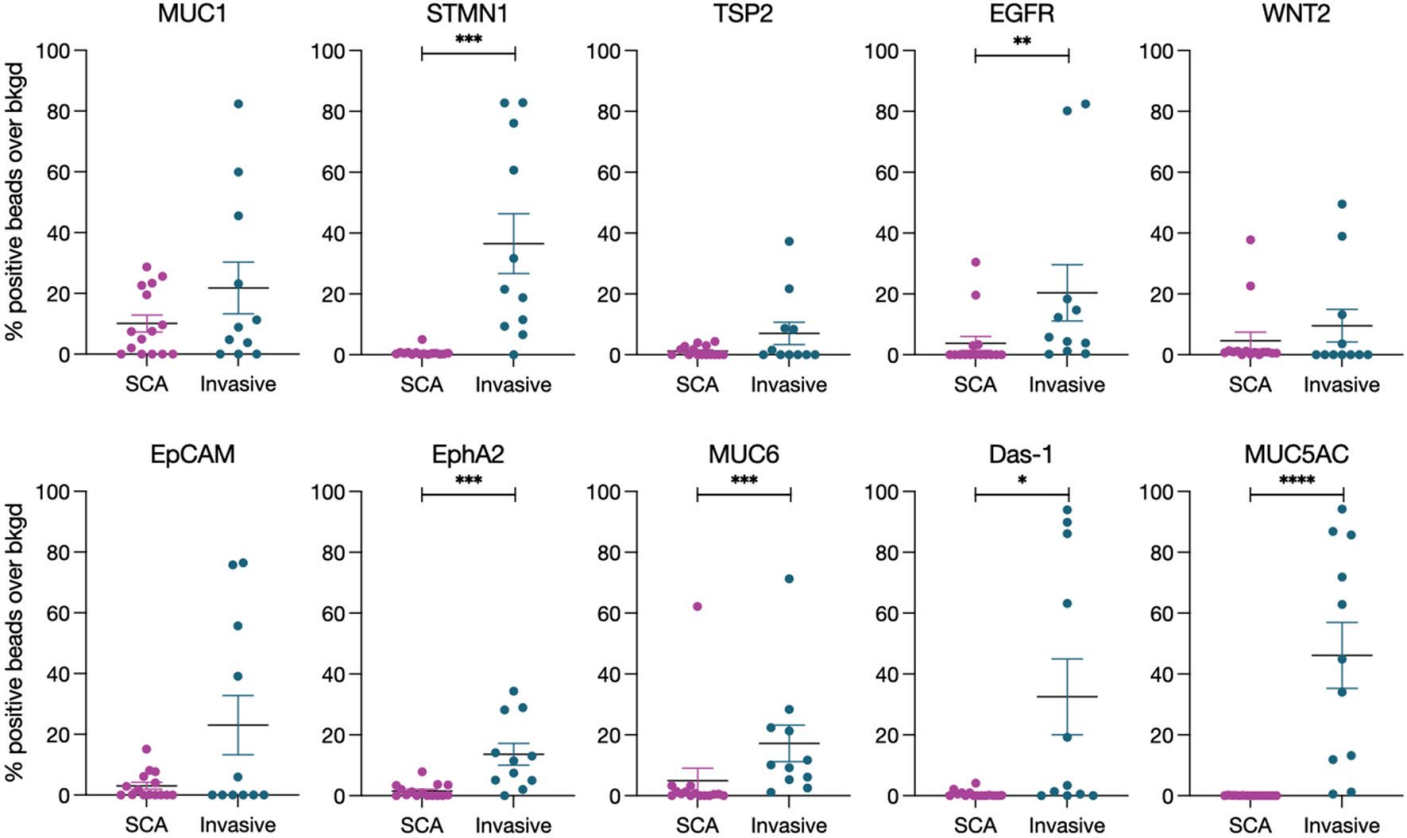
EV analysis allows the detection of invasive cancers in cystic lesions. SCA (purple; all cases of typical and atypical SCA by imaging) plasma EV were compared to invasive IPMN (blue) using the indicated biomarkers. Error bars represent standard error of the mean. *P*-values < 0.05 (*) were calculated using a Mann–Whitney test.

While no signature emerged for the combined group of all SCAs, we next asked whether malignant degeneration could be detected in plasma samples from patients with any cystic lesion (Fig. 2). Samples from patients with SCAs (n = 15) and with invasive IPMNs (n = 11) were thus also screened for 9 previously established high-grade IPMN biomarkers MUC5AC, Das-1, MUC1, STMN1, TSP2, EGFR, WNT-2, EpCAM, and EphA2^13^. Invasive IPMNs demonstrated significantly higher expression as compared to SCAs (including atypical ones) for the following biomarkers MUC5AC (46.1 ± 10.9% vs. 0.04 ± 0.02%, *P* < 0.0001), STMN1 (36.6 ± 9.8% vs. 0.74 ± 0.3%, *P* = 0.0002), EGFR (20.4 ± 9.3% vs. 3.8 ± 2.3%, *P* = 0.0011), EphA2 (13.6 ± 3.6% vs. 1.6 ± 0.6%, *P* = 0.0005), MUC6 (17.2 ± 6% vs. 4.9 ± 4.1%, *P* = 0.0002), and Das-1 (32.5 ± 12.5% vs. 0.6 ± 0.3%, *P* = 0.03) (Fig. 2).

### Biomarkers in SCA subgroups

**Figure 3:**
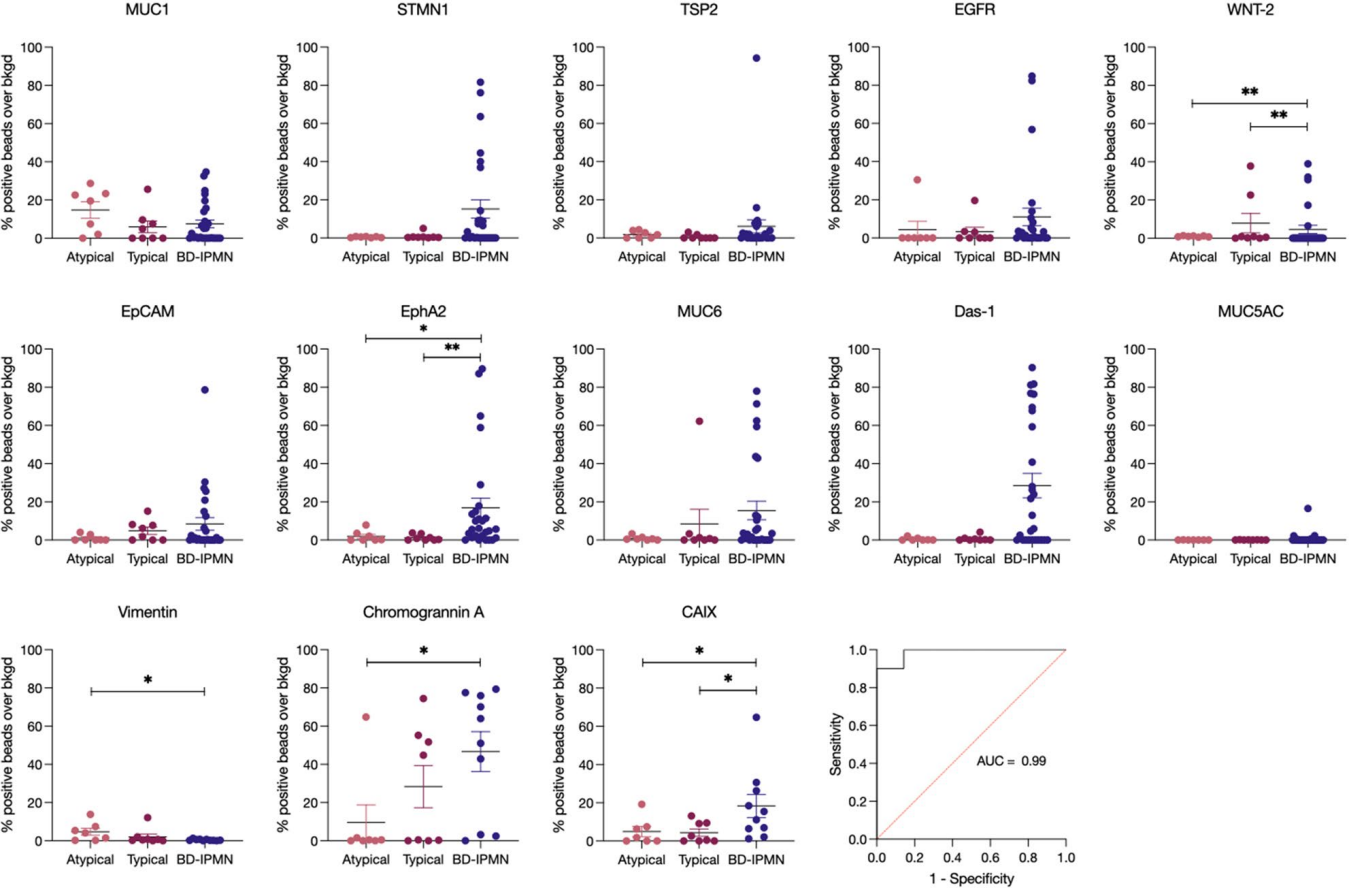
Biomarker expression in atypical and typical SCA compared to BD-IPMN. Atypical (light pink) and typical (dark pink) SCA compared to BD-IPMN (dark purple) for malignant and benign biomarkers. Error bars represent standard error of the mean. *P*-values (*, < 0.05) were determined using a Mann–Whitney test. Multiple logistic regression analysis of atypical versus BD-IPMN using a four-way biomarker combination of Das-1, Vimentin, Chromogranin A, and Carbonic Anhydrase I × (CAIX) is shown in the bottom right ROC curve. The AUC was determined using Prism.

To identify whether biomarkers would be present in typical and atypical SCA subgroups, we sub-categorized the SCA samples into typical and atypical ones based on their findings by MRI. Since atypical SCA can have imaging features of branch-duct (BD) IPMNs we compared to this cohort. Figure 3 shows the comparison of EV analysis from the three groups. Most biomarkers showed higher expression in BD-IPMN compared to atypical and typical SCA, respectively: MUC1 (7.5 ± 2.1% vs. 14.8 ± 4.3% vs. 5.9 ± 3.1%), STMN1 (15.2 ± 4.7% vs. 0.5 ± 0.1% vs. 1.0 ± 0.6%), TSP2 (6.1 ± 3.5% vs. 1.8 ± 0.7% vs. 0.6 ± 0.4%), EGFR (11.0 ± 4.6% vs. 4.3 ± 4.3% vs. 3.2 ± 2.4%), WNT-2 (4.6 ± 2.1% vs. 1.0 ± 0.2% vs. 7.8 ± 5.1%), EpCAM (8.4 ± 3.3% vs. 1.0 ± 0.6% vs. 4.9 ± 1.9%), EphA2 (16.9 ± 5.0% vs. 1.9 ± 1.1% vs. 1.2 ± 0.6%), MUC6 (15.4 ± 4.8% vs. 0.9 ± 0.4% vs 8.4 ± 7.7%), Das-1 (28.5 ± 6.4% vs. 0.5 ± 0.3% vs. 0.7 ± 0.5%), MUC5AC (0.8 ± 0.6% vs. 0.02 ± 0.02% vs. 0.06 ± 0.03%), Chromogranin A (46.7 ± 10.4% vs. 9.6 ± 9.2% vs. 28.4 ± 11.0%), and CAIX (18.3 ± 6.0% vs. 5.0 ± 2.6% vs. 4.4 ± 1.9%). The notable exception was Vimentin, which showed slightly higher levels in atypical SCA (0.4 ± 0.1% vs. 4.7 ± 1.8% vs. 2.0 ± 1.5%, *P* = 0.0229 for atypical vs. BD-IPMN). We next used univariate analysis and then multivariate analysis of atypical SCA versus BD-IPMN. Analysis of this data showed that a four-way biomarker combination of Das-1, Vimentin, Chromogranin A, and CAIX had a ROC area under the curve (AUC) score of 0.99, which suggested a strong (*P* = 0.0009) discriminative capability for discerning between BD-IPMNs requiring surgery and atypical SCAs not requiring surgery (Fig. 3). A limitation of this study is the small and exploratory nature of the biomarker signature, which was derived from only 15 SCA samples. Therefore, the actual performance of the signature in discriminating SCA from other conditions may be lower than reported here.

### Comparison of EV obtained from plasma and cyst fluid

**Figure 4:**
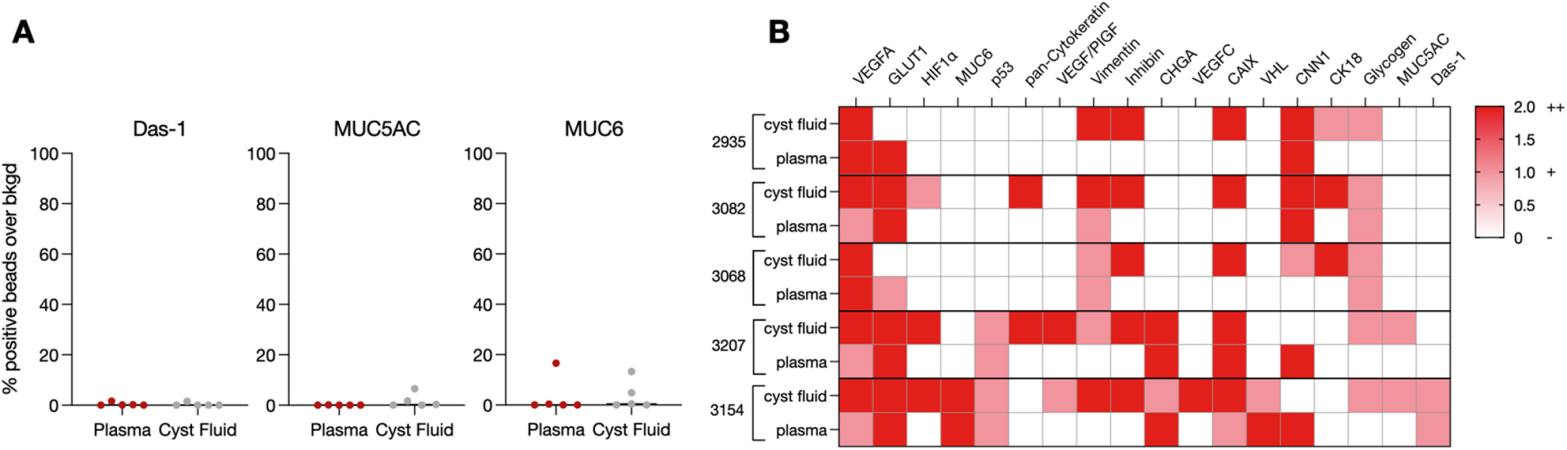
SCA DEST plasma correlation with cyst fluid. Biomarkers were measured in matched SCA plasma and cyst fluid samples from five patients. (**A**) Malignant biomarkers are at low expression in both plasma and cyst fluid. (**B**) SCA biomarker correlation between matched plasma and cyst fluid samples.

In five subjects, cyst fluid-matched samples from aspirated SCA and plasma were obtained. DEST analysis for EV in these matched samples demonstrated low plasma and cyst fluid expression for the malignant biomarkers Das-1, MUC5AC, and MUC6 (Fig. 4A, Fig. S5). In contrast, the SCA biomarker VEGFA was highly expressed in cyst fluid and plasma (Fig. 4B, Fig. S5). Of the remaining SCA biomarkers tested, plasma and cyst fluid EV correlate in half of the biomarkers tested, with CAIX levels the least correlative. Vimentin expression is overall low in both plasma and cyst fluid EV. Levels of some biomarkers were too low in circulating plasma to be reliably detected (Fig. 4B, Fig. S5). While this data is limited to a small number of patients, it suggests that EV obtained from cyst fluid are likely not superior in diagnostic performance compared to plasma EV.

## Discussion

Serous cystadenomas are considered benign pancreatic cysts that do not require surgical intervention unless symptomatic. Unfortunately, up to 20% of SCAs have atypical imaging findings that trigger expensive, lengthy, and invasive follow-ups. A liquid biopsy test would thus be instrumental to better categorize cystic lesions. This could be a test to either positively define lesions that do not require further work-up or identify those that require surgery. We argued that plasma-derived EV analysis could form the basis of tests based on prior research in identifying early-stage cancers^11–13,27^. EVs are < 200 nm membrane-bound structures released by cells and generally contain a variety of biomolecules, including proteins, lipids, and nucleic acids from their parental cells of origin. The goal of the current study was to establish EV biomarker tests that are clinically practical, accurate, and cost-effective. We developed a new DEST assay and validated it for 25 EV-associated protein biomarkers. Once developed and validated, we performed measurements on biobank samples that had been carefully established, curated, and annotated with clinical information.

Our data show that no single plasma EV biomarker uniquely identified all SCA. It is possible that such protein biomarkers indeed exist but have not been identified to date. Research in this field will likely require proteomic analysis and perhaps require biomarker measurement at the single EV level, a field that is currently not mature enough. Conversely, there may not be any discernible SCA-derived unique EV at all, or at least in quantities sufficient enough for current detection technologies. Most recently, it has become possible to perform multiplexed single EV analysis^27^, which could potentially be applied to SCA samples in more depth. In initial feasibility experiments, however, we are not able to pinpoint defining biomarkers.

Given the above, we thus focused on biomarkers that would identify cystic lesions that warrant surgical resection, i.e., potentially harboring malignant transformations. In the present study, we identified a unique EV signature that distinguishes between cystic lesions with invasive cancers requiring surgery and atypical SCA that do not. Given the overlap in imaging appearances of these two entities but drastically different management, this represents a promising step towards early blood-based screening for triage of these lesions in clinical practice. The diagnostic performance of a multivariable regression model for discriminating between lesions that require resection and those that do not was excellent, with an AUC of 0.99.

In atypical cases with diagnostic uncertainty about the exact etiology of a cystic pancreatic neoplasm, further evaluation with endoscopic ultrasound (EUS) and cyst sampling has been recommended^28^, but is also fraught with limitations. Elevated cyst CEA levels are more commonly seen in MCNs and IPMNs^7^, and up to a third of these can have low or very low cyst fluid CEA. Although CEA is almost universally low^19^, the presence of serous epithelial cells used to affirm the diagnosis of SCAs is seen in less than 20% of specimens^29^. VEGF in fluid or a mutated VHL gene has also been reported in small series^30,31^. Furthermore, contamination from gastrointestinal epithelium in the sample confounds cytopathologic analysis^29^. We were able to compare EV in cyst fluid and plasma from the same patient in five cases. Our data show that plasma EV properties generally matched those of cyst EV for half the biomarkers studied.

While the reported methodological advances and clinical results are promising, our study had some caveats that may require follow-up studies. First, our study was based on a retrospective analysis of banked samples rather than occurring as a prospective study. This was done intentionally because we relied on clinical annotations of biobank samples to ensure that patients didn't progress to malignant lesions years after sampling. Second, our sample numbers are modest and future studies may necessitate pooling of samples from different institutions to increase study power. Third, our assay relies on commercial antibodies. While we screened for hundreds of antibodies from other vendors, it is possible that future antibody clones may be superior to the ones tested here. Irrespective of these limitations and caveats, our preliminary data affirm the potential utility of EV in cystic pancreatic neoplasms as a diagnostic tool.

## Materials and methods

### Study group

This study was approved by the institutional review board of Massachusetts General Hospital, and written informed consent was obtained from study participants. All experiments were conducted in accordance with the IRB guidelines and regulations. Specimens were collected from patients referred to Massachusetts General Hospital for surgical management. Participant details can be found in Fig. S3. The biobank has been described and is continually being updated^3^.

### EV biomarkers

Based on a literature review in 2021 using Pubmed and Cited Reference Search (Web of Science; search terms: pancreatic cyst fluid, proteomics, serous cystadenoma, intraductal papillary mucinous neoplasm, biomarkers, pancreatic cysts), we selected putative biomarkers of SCA and other cystic pancreatic lesions, which might potentially be useful to discriminate between benign and malignant pancreatic cystic lesions. Since there are no prospective nor proteomic analyses on SCA samples, we focused primarily on biomarkers that had been identified in pathology or cyst fluid studies^15–24^. We were agnostic to the accuracy of any of the biomarkers but validated each one in cell lines prior to the measurement of human plasma (see below).

### Antibody-bead coupling and biotinylation

Capture antibodies (Table 1) were coupled to dynabeads M-270 Epoxy magnetic beads using a coupling kit (Thermo, 14311D), with buffers supplied in the kit (Buffers C1, C2, HB, LB, SB). Dynabeads were weighed into Eppendorf tubes and washed once with Buffer C1. Capture antibody was mixed with the beads at a ratio of 10 μg antibody/mg dynabeads. Buffer C1 was mixed with the antibody solution (50 μL/mg bead). The total reaction volume (Buffer C1 + antibody + Buffer C2) was 100 μl per mg dynabeads. The bead-antibody mixture was incubated overnight at 37 °C on a HulaMixer (35 rpm, 5° tilt, 5° rotation; all 5 s). Coupling efficiency was determined using the supernatant after bead coupling. Beads were washed with buffer HB and LB (containing 0.05% Tween-20), followed by two washes with buffer SB, and then incubation in buffer SB for 15 min on a HulaMixer. The solution was removed and the final antibody-bead conjugate was stored at 4 °C in 100 μL buffer SB/mg dynabead. After each wash, beads were incubated 1 min on a DynaMag magnet and wash buffer was discarded.

**Table 1:** Antibodies used in the DEST assay.

Target	Capture Antibody			Detection Antibody		
	Vendor	Cat No	Clone	Vendor	Cat No	Clone
VEGFA	BioLegend	537002	A15136H	BioLegend	522503	Poly5225
GLUT1	Abnova	RAB00625	GLUT1/3132R	Sigma	07-1401	polyclonal
HIF1α	R&D	DYC1935-2	polyclonal	R&D	DYC1935-2	polyclonal
MUC6	MyBioSource	MBS2003026	polyclonal	MyBioSource	MBS2055835	polyclonal
p53	R&D	DYC1043-2	polyclonal	R&D	DYC1043-2	polyclonal
pan-Cytokeratin	GeneTex	GTX48825	C-11	Santa Cruz	sc-8018 HRP	C11
VEGF/PIGF	R&D	DY297-05	polyclonal	R&D	DY297-05	polyclonal
Vimentin	GeneTex	GTX629744	GT812	GeneTex	GTX100619	polyclonal
Inhibin	R&D	MAB338	130408R	R&D	AF10024	polyclonal
Chromogranin A	R&D	DY9098-05	polyclonal	R&D	DY9098-05	polyclonal
VEGFC	R&D	DY752B	polyclonal	R&D	DY752B	polyclonal
CAIX	R&D	DY2188	polyclonal	R&D	DY2188	polyclonal
VHL	GeneTex	GTX101087	polyclonal	GeneTex	GTX89268	polyclonal
CNN1	Abnova	H00001264-AP41	polyclonal	Abnova	H00001264-AP41	polyclonal
Cytokeratin 18	Novus	NB500-306	C-04	GeneTex	GTX78239	DC-10
Glycogen (STBD1)	Novus	NBP2-22828	n/a	Abnova	H00008987-Q02	n/a
MUC1	Fitzgerald	10-CA15A	M201211	Fitzgerald	10-CA15B	M2012112
STMN1	Rockland	600-401-DG7	polyclonal	Rockland	600-401-DG8	polyclonal
TSP2	R&D	MAB16351	230927	R&D	BAF1635	polyclonal
EGFR	R&D	AF231	polyclonal	Abcam	ab98133	polyclonal
WNT2	MyBioSource	2104322	polyclonal	MyBioSource	2104322	polyclonal
EpCAM	R&D	MAB9601	158206	R&D	BAF960	polyclonal
EphA2	R&D	MAB3035	371805	R&D	BAF3035	polyclonal
Das-1	Millipore	MABC530	7E12H12	Millipore	MABC530	7E12H12
MUC5AC	Abcam	ab24070	1-13M1	Thermo	MA5-12175	45M1

Capture and detection antibody vendors and antibody clones are indicated. n/a = not applicable.

Detection antibodies (Table 1) that were not readily available as a biotinylated or HRP-conjugated product, were prepared using sulfo-NHS-LC-Biotin (Thermo, A39257). Briefly, a 20-fold molar excess of biotin was calculated for 50 μg antibody. 180μL ultrapure water was added to a 1 mg no-weigh vial of biotin to make a 10 mM stock solution. The calculated volume of biotin was added to the antibody in PBS and incubated for 30 min at room temperature. Excess biotin was removed using a 0.5 mL, 7MWCO Zeba column (Thermo, 89882). Biotinylated antibody concentrations were determined using a Nanodrop (Thermo, ND-1000), and antibodies were stored under the same conditions as the unmodified antibody.

### DEST assay

Table S1 outlines the steps and incubation times of the DEST assay. Table 2 lists the reagents needed for the assay. Beads were measured on a CytoFlex flow cytometer (Beckman Coulter) with the following settings: FSC 201 V, SSC 90 V, PB450 (BV421 detection) 40 V. Single bead gates were drawn on the FSC-A versus SSC-A dot plot, and 10,000 events were recorded for each replicate. Data analysis was done using FlowJo (BD, version 10.8.1). Pooled normal human plasma served as a background control, and a gate was drawn using the bisector tool to delineate positive from negative beads. All data are reported as the percentage of positive beads out of 10,000 total beads and are the average of duplicate measurements. Error is represented as the standard error of the mean.

**Table 2:** List of reagents used in the DEST assay.

Reagent	Company	Catalog #	Stock conc	Final conc
Bovine serum albumin	Fisher Scientific	BP1605-100	–	2% w/v
UltraBlock	Bio-Rad	BUF033C	–	10–100%
ELISA general assay diluent	Bio-Rad	BUF037A	–	use neat
HAMA Blocker	Abcam	ab193969	–	use neat
PBS	Thermo Scientific	70011069	10X	1X
Tween-20	Sigma-Aldrich	P9416-100 mL	100%	0.1%
Pooled normal human plasma (K2 EDTA)	Innovative Research Inc	IPLA-N	–	1-10 μl
Streptavidin-HRP	Thermo Scientific	21130	1.1 mg/mL	137.5 ng/mL
Biotinyl tyramide	Sigma-Aldrich	SML2135-50 mg	2 mg/mL (DMSO)	5 μg/mL
Borate buffer	Thermo Scientific	28341	20X	2X
Hydrogren peroxide solution	Sigma-Aldrich	H1009-100 mL	30%	0.003%
Brilliant violet 421 streptavidin	BioLegend	405225	0.5 mg/mL	0.5 μg/mL

### Cell culture

1505 and 1966 IPMN PDX and 1617 PDAC PDX cell lines were from the MGH pancreas biobank^32^ and were maintained in a 50:50 mix of Ham’s F-12 and Dulbecco’s modified eagle’s medium. 1617 cells were supplemented with 10% FBS and 100 IU penicillin/100 μg/ml streptomycin (Mediatech 30-002-CI), while IPMN PDX lines were supplemented with 20% fetal bovine serum (FBS), 1% antibiotic–antimycotic (Thermo, 15240062), 10 mM Nicotinamide (Sigma-Aldrich, N0636), 1X insulin-transferrin-selenium (Corning, 25-800-CR), 8.4 ng/mL cholera toxin (Sigma-Aldrich, C8052), 10 ng/mL epidermal growth factor (Sigma-Aldrich, E9644), and 10 ng/mL hepatocyte growth factor (Thermo, PHG0324). Other cell lines used as positive or negative controls were grown according to ATCC in media with 10% FBS and 100 IU penicillin/100 μg/ml streptomycin (Mediatech 30-002-CI) and include: Daudi (CCL-213), U-87 MG (HTB-14), MCF7 (HTB-22), Capan-2 (HTB-80), A-431 (CRL-1555), Mia PaCa-2 (CRM-CRL-1420), BxPC-3 (CRL-1687), A549 (CCL-185), and LS 180 (CL-187).

### EV isolation from cell culture

In order to test different antibodies and experimental conditions prior to clinical use, we harvested EV from the culture of cell lines listed above. Cells were grown for 48-72 h in normal growth medium supplemented with 5% exosome-depleted FBS (Thermo, A2720801). Conditioned media was collected and centrifuged at 300 × g for 10 min to remove dead cells and debris, followed by filtration through a 0.22-μm cellulose acetate vacuum filter (Corning, 430767). Media was then concentrated to ~ 1 mL using a Centricon Plus-70 Centrifugal Filter (Millipore Sigma, UFC710008) and centrifugation at 4000 × g for 20 min, following the protocol of Lobb et al.^33^. EV were then purified from the concentrated media using a qEV original column from IZON (iZON Science, SP1) following the manufacturer’s instructions.

### Clinical sample processing

Blood collection for plasma EV analysis was done as described in Lobb et al.^33^. All samples were de-identified and analyzed in a blinded fashion. Briefly, whole blood was collected in a 10-mL purple-top K2-EDTA tube, inverted 10 times to mix, then stored upright at 4 °C and processed within 1 h of collection. Plasma isolation was then done by centrifugation for 10 min at 400 × g (4 °C). The plasma layer was collected in a 15 mL tube using a pipette without disturbing the buffy coat. A second centrifugation step was done for 10 min at 1100 × g (4 °C). The plasma was then aliquoted to 1 mL and stored at − 80 °C.

### Plasma preparation for DEST

Plasma was thawed at 4 °C and then lysed in 1 × RIPA buffer (Cell Signaling Technology, 9806S, 6 × stock) for 15 min on ice to allow for both surface and intravesicular biomarker profiling on EV. Lysed plasma was aliquoted and stored at − 80 °C until use.

### Statistics

All statistical data analyses were performed using GraphPad Prism 9 (Dotmatics, Boston) software. Results are expressed as mean ± standard error. Significance between groups was determined using a Mann–Whitney unpaired, nonparametric two-tailed t-test. Multiple logistic regression was used to determine the best biomarker combination to distinguish atypical SCA from BD-IPMN.

## Supplementary Information

Supplementary Information.

## Data Availability

Data is available on request from the corresponding author.
