# Supplementary material for: Extracellular vesicle analysis of plasma allows differential diagnosis of atypical pancreatic serous cystadenoma
Source: Sci Rep. 2023 Jul 6;13:10969. doi: 10.1038/s41598-023-37966-5 (PMC10325992; doi:10.1038/s41598-023-37966-5)
Supplement: Supplementary file 1 — Supplementary Information. [file 41598_2023_37966_MOESM1_ESM.docx]

**Supplementary Materials**

**Extracellular vesicle analysis of plasma allows differential diagnosis of atypical pancreatic serous cystadenoma**

Katherine S. Yang^1,2^, Aileen O’Shea^1,2^, Piotr Zelga^3^, Andrew S. Liss^3^, Carlos Fernandez del Castillo^3^, Ralph Weissleder^1,2,4^*


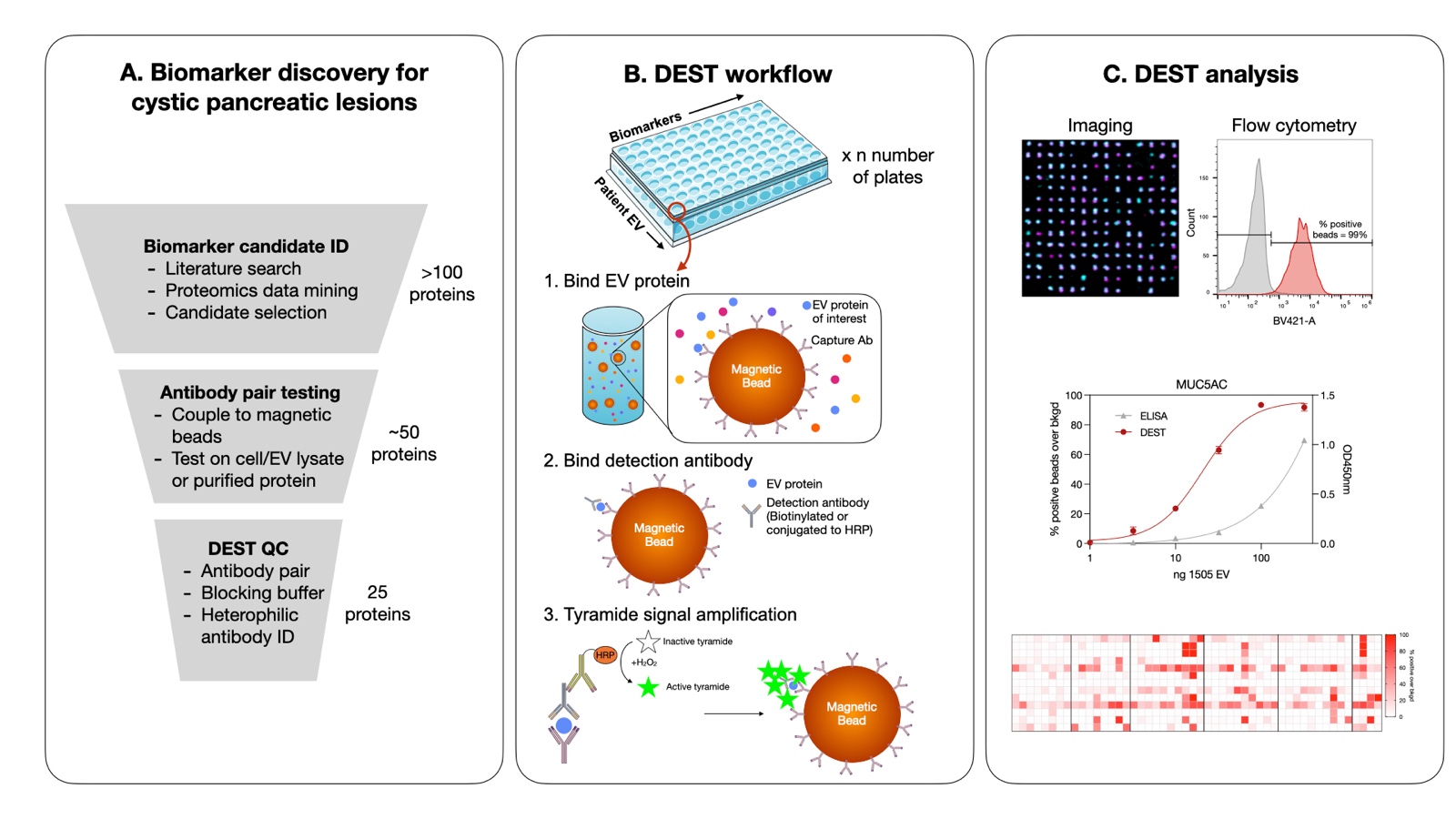
**Fig. S1: DEST assay.** A) Putative biomarkers were selected by an extensive literature search. Candidate biomarkers were further refined based on antibody pair availability. DEST QC to test and optimize the biomarker pairs further refined the panel to 25 measurable proteins in EV. B) The DEST workflow utilizes a 96-well plate matrix with patient EV and biomarker-specific antibody-coated magnetic beads to rapidly and efficiently measure the biomarker panel in patient plasma samples. Three main steps encompass the DEST assay: 1) Magnetically separate EV proteins of interest, 2) Binding of detection antibody, and 3) tyramide signal amplification. C) DEST readout can be measured by imaging or flow cytometry. The assay displays increased sensitivity compared to traditional ELISA.

**Fig. S2: DEST assay validation.** SCA and invasive biomarkers were validated in the
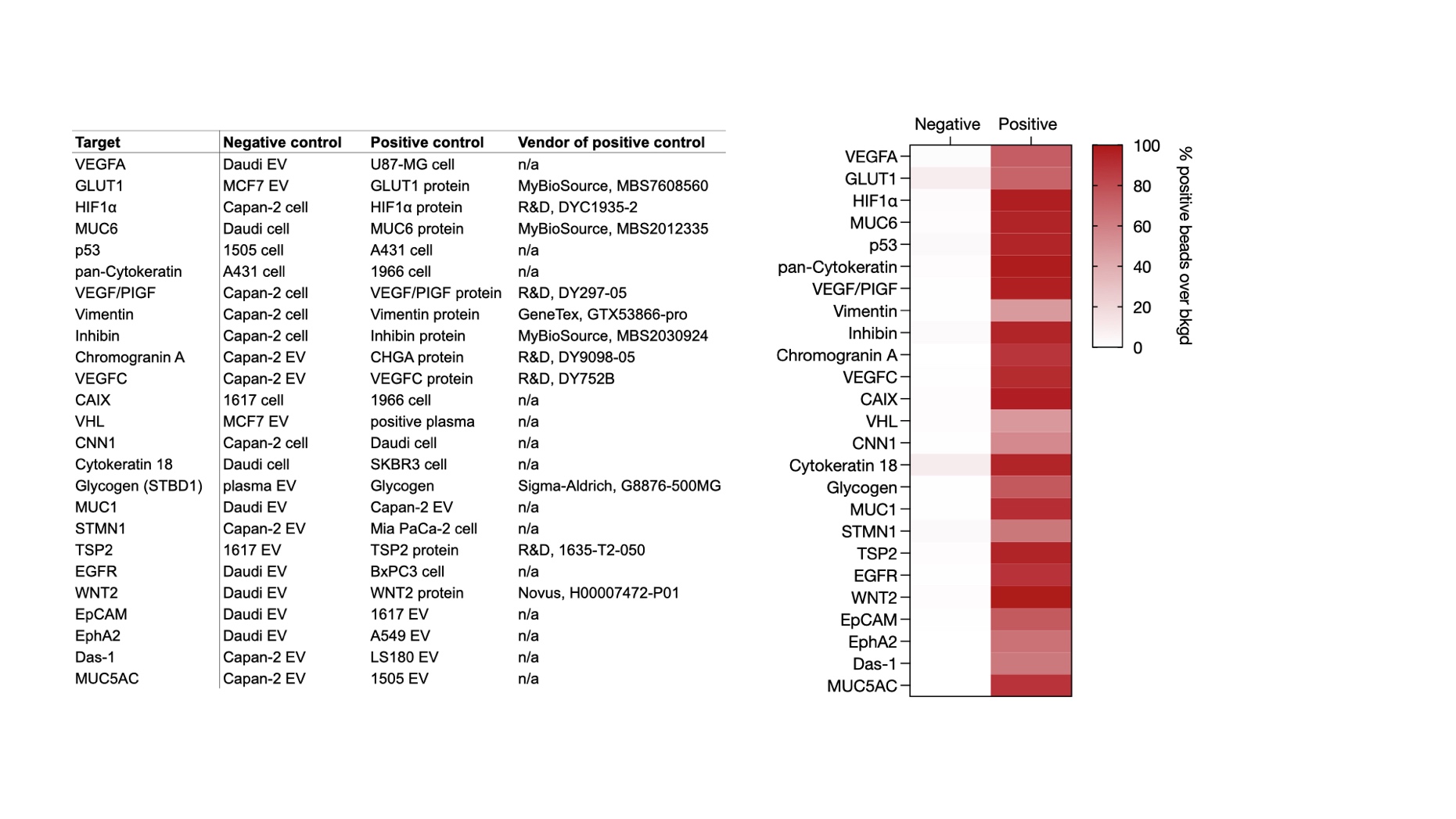
DEST assay using EV, cell lysates, and purified protein.

**Fig. S3: Patient cohorts and demographics.**


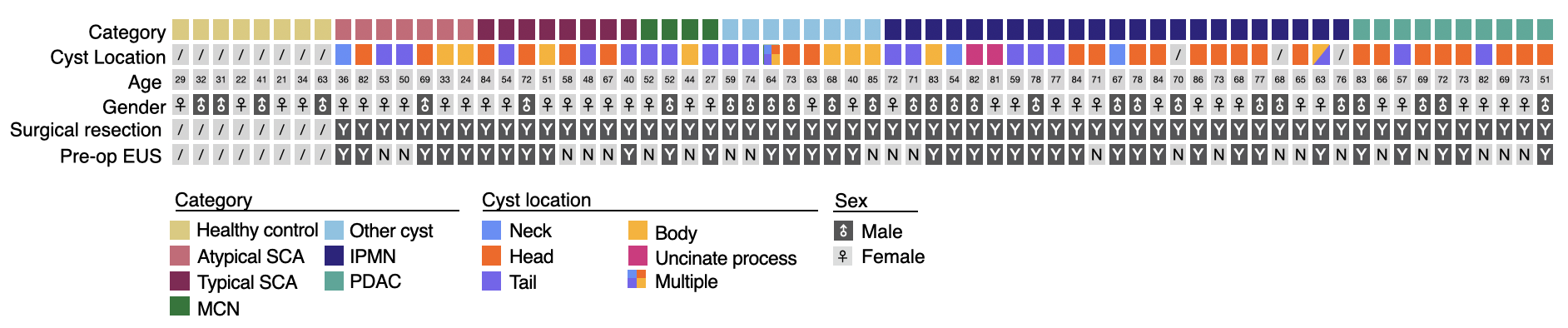
A total of 68 patients were enrolled in the study, all of whom had surgical resection (except for controls). The cohorts included atypical SCAs (n=7), typical SCAs (n=8), IPMNs (n=23), MCNs (n=4), other cysts (n=8), pancreatic adenocarcinoma (n=10), and healthy controls (n=8).

**Fig. S4: SCA biomarker expression in all pathologies.** Putative SCA biomarkers were tested in a cohort of samples that included healthy controls, SCA, other cysts, MCN, LG- and HG-IPMN, and PDAC. Patient sample details can be found in **Fig. S3**. Error bars represent standard error of the mean, and p-values (* p-value < 0.05) were calculated using a Mann-Whitney test.

**Fig. S5: Comparison of plasma and cyst fluid analysis.** Select biomarkers were compared between matched plasma and cyst fluid samples (n = 5) using the DEST assay. Plasma and cyst fluid levels indicated similar positivity/negativity for the majority
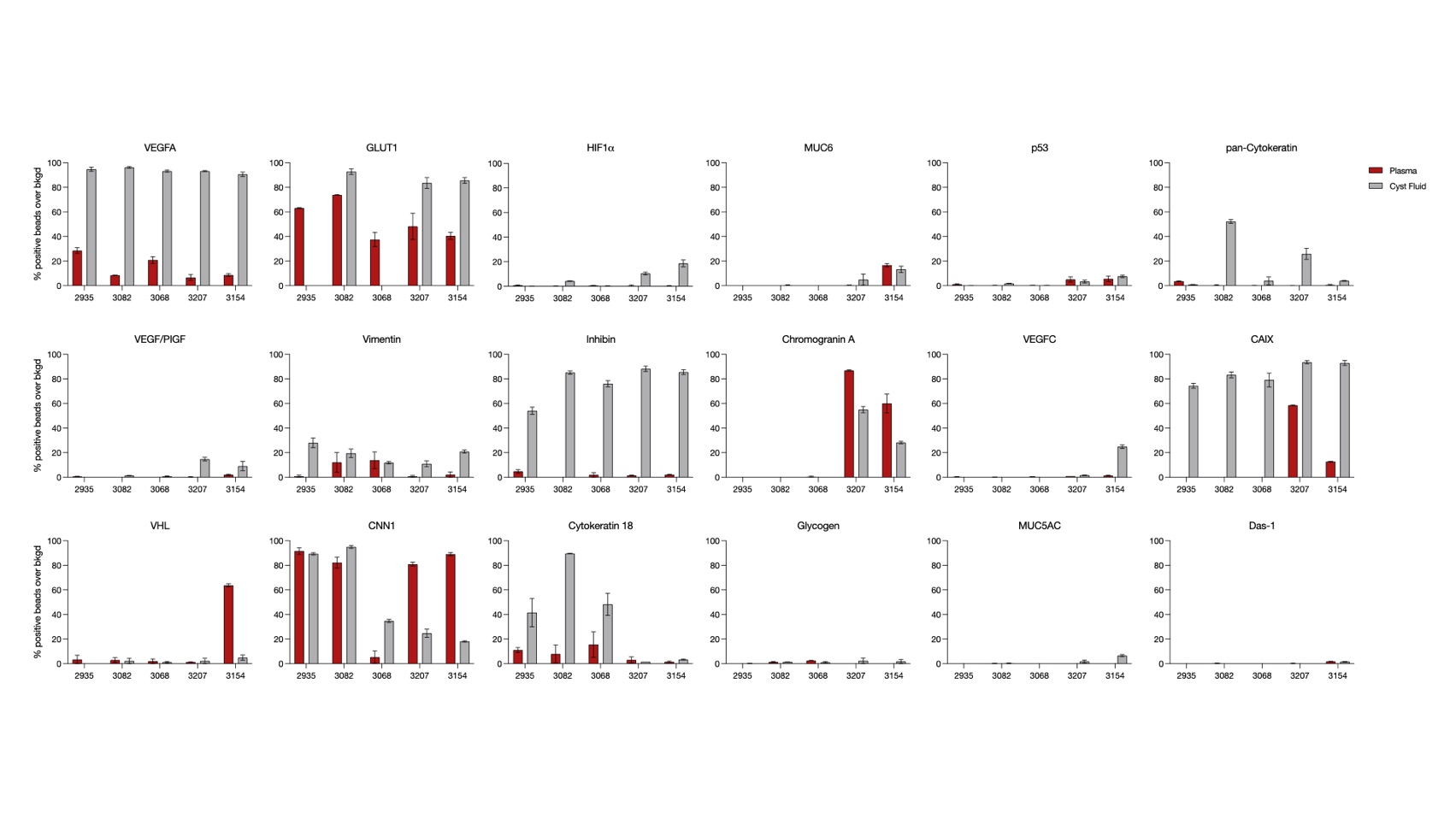
of biomarkers measured, with the exception of Inhibin and CAIX.

**Table S1: DEST assay details.** 10 µL of each plasma EV sample is used per replicate. Beads are collected using a 96-well plate magnet for each wash step. Each incubation step is done on a plate shaker to maintain beads in suspension. MUC1 and MUC5AC were incubated in HAMA blocker in step 3.

| **Step** | **Reagent** | **Time (min)** | **Vol. (μL)** | **Conc.** | **Buffer** |
| --- | --- | --- | --- | --- | --- |
| 1 | Blocking | 15 | 100 | 1.5μl  (~1 million) beads/well | Antibody dependent, see Table S2 |
| 2 | Wash | 4 washes | 100 | — | PBS + 0.1% Tween-20 |
| 3 | Incubation with sample | 45 | 100 | 10μl plasma, 500ng EV or cell lysate controls | Blocking buffer (Table S4) |
| 4 | Wash | 3 washes | 100 | — | PBS + 0.1% Tween-20 |
| 5 | Detection antibody | 30 | 50 | see Table S4 | Blocking buffer |
| 6 | Wash | 3 washes | 100 | — | PBS + 0.1% Tween-20 |
| 7 | Streptavidin-HRP | 30 | 100 | 137.5ng/mL | Blocking buffer + 0.1% Tween-20 |
| 8 | Wash | 3 washes | 100 | — | PBS + 0.1% Tween-20 |
| 9 | Biotin tyramide (signal amplification) | 10 | 100 | 5μg/mL | 0.1M Borate buffer (pH8.5) + 0.003% H2O2 |
| 10 | Wash | 3 washes | 100 | — | PBS + 0.1% Tween-20 |
| 11 | Brilliant violet 421 streptavidin | 30 | 50 | 0.5μg/mL | Blocking buffer |
| 12 | Wash | 3 washes | 100 | — | PBS + 0.1% Tween-20 |
| **Total** |  | **160 minutes** |  |  |  |

**Table S2:** **DEST antibody pair conditions.** Blocking buffers used for each antibody pair.

| **Target** | **Blocking buffer** | **DEST step 3 buffer** |
| --- | --- | --- |
| **VEGFA** | 2% BSA in PBS | 2% BSA in PBS |
| **GLUT1** | 2% BSA in PBS | ELISA general assay diluent |
| **HIF1α** | 2% BSA in PBS | ELISA general assay diluent |
| **MUC6** | 2% BSA in PBS | 2% BSA in PBS |
| **p53** | 2% BSA in PBS | 2% BSA in PBS |
| **pan-Cytokeratin** | 2% BSA in PBS | 2% BSA in PBS |
| **VEGF/PIGF** | 2% BSA in PBS | 2% BSA in PBS |
| **Vimentin** | 2% BSA in PBS | 2% BSA in PBS |
| **Inhibin** | 2% BSA in PBS | 2% BSA in PBS |
| **Chromogranin A** | UltraBlock | UltraBlock |
| **VEGFC** | 10% UltraBlock in PBS | 10% Ultrablock in PBS |
| **CAIX** | 2% BSA in PBS | ELISA general assay diluent |
| **VHL** | 2% BSA in PBS | 2% BSA in PBS |
| **CNN1** | 2% BSA in PBS | ELISA general assay diluent |
| **Cytokeratin 18** | 2% BSA in PBS | ELISA general assay diluent |
| **Glycogen (STBD1)** | 2% BSA in PBS | 2% BSA in PBS |
| **MUC1** | 2% BSA in PBS | 2% BSA in PBS, HAMA blocker |
| **STMN1** | 2% BSA in PBS | 2% BSA in PBS |
| **TSP2** | 2% BSA in PBS | 2% BSA in PBS |
| **EGFR** | 2% BSA in PBS | 2% BSA in PBS |
| **WNT2** | 2% BSA in PBS | 2% BSA in PBS |
| **EpCAM** | 2% BSA in PBS | 2% BSA in PBS |
| **EphA2** | 2% BSA in PBS | 2% BSA in PBS |
| **Das-1** | UltraBlock | UltraBlock |
| **MUC5AC** | UltraBlock | UltraBlock, HAMA blocker |
